# Low Serum Levels of DKK2 Predict Incident Low‐Impact Fracture in Older Women

**DOI:** 10.1002/jbm4.10179

**Published:** 2019-03-28

**Authors:** Ana M Rodrigues, Mónica Eusébio, Ana B Rodrigues, Joana Caetano‐Lopes, Inês P Lopes, Ana Lopes, Jorge M Mendes, Pedro Simões Coelho, João Eurico Fonseca, Jaime C Branco, Helena Canhão

**Affiliations:** ^1^ CEDOC EpiDoc Unit–Epidemiology of Chronic Diseases Nova Medical School Universidade Nova de Lisboa Lisboa Portugal; ^2^ Faculdade de Medicina da Universidade de Lisboa Lisboa Portugal; ^3^ Associação EpiSaúde Évora Portugal; ^4^ Sociedade Portuguesa de Reumatologia Lisboa Portugal; ^5^ Department of Orthopaedic Research Boston Children's Hospital, Boston, MA, USA; Department of Genetics Harvard Medical School Boston MA USA; ^6^ Unidade de Investigação em Reumatologia Instituto de Medicina Molecular Faculdade de Medicina Universidade de Lisboa Centro Académico de Medicina de Lisboa Lisboa Portugal; ^7^ NOVA IMS Universidade Nova de Lisboa Lisboa Portugal; ^8^ Serviço de Reumatologia e Doença Ósseas Metabólicas Hospital de Santa Maria CHLN Centro Académico de Medicina de Lisboa Lisboa Portugal; ^9^ Centro de Estudos de Doenças Crónicas (CEDOC) da NOVA Medical School Universidade Nova de Lisboa (NMS/UNL) Lisboa Portugal; ^10^ Serviço de Reumatologia do Hospital Egas Moniz–Centro Hospitalar Lisboa Ocidental (CHLO‐ E.P.E.) Lisboa Portugal; ^11^ Escola Nacional de Saúde Pública Universidade Nova de Lisboa Lisboa Portugal

**Keywords:** FRACTURE RISK ASSESSMENT, SCREENING, MOLECULAR PATHWAYS–REMODELING, Wnt/β‐CATENIN/LRPs, AGING

## Abstract

There are currently no robust noninvasive markers of fragility fractures. Secreted frizzled related protein‐1 (sFRP‐1), dickkopf‐related protein 1 (DKK1) and DKK2, and sclerostin (SOST) inhibit Wnt signaling and interfere with osteoblast‐mediated bone formation. We evaluated associations of serum levels of sFRP‐1, DKK1, DKK2, and SOST with incident low‐impact fracture and BMD in 828 women aged ≥65 years from EpiDoC, a longitudinal population‐based cohort. A structured questionnaire during a baseline clinical appointment assessed prevalent fragility fractures and clinical risk factors (CRFs) for fracture. Blood was collected to measure serum levels of bone turnover markers and Wnt regulators. Lumbar spine and hip BMD were determined by DXA scanning. Follow‐up assessment was performed through a phone interview; incident fragility fracture was defined by any new self‐reported low‐impact fracture. Multivariate Cox proportional hazard models were used to analyze fracture risk adjusted for CRFs and BMD. During a mean follow‐up of 2.3 ± 1.0 years, 62 low‐impact fractures were sustained in 58 women. A low serum DKK2 level (per 1 SD decrease) was associated with a 1.5‐fold increase in fracture risk independently of BMD and CRFs. Women in the two lowest DKK2 quartiles had a fracture incidence rate of 32 per 1000 person‐years, whereas women in the two highest quartiles had 14 fragility fractures per 1000 person‐years. A high serum sFRP1 level was associated with a 1.6‐fold increase in fracture risk adjusted for CRFs, but not independently of BMD. Serum levels of SOST (*r* = 0.191; *p* = 0.0025) and DKK1(*r* = −0.1725; *p* = 0.011) were correlated with hip BMD, but not with incident fragility fracture. These results indicate that serum DKK2 and sFRP1 may predict low‐impact fracture. The low number of incident fractures recorded is a limitation and serum levels of Wnt regulators should be further studied in other populations as potential noninvasive markers of fragility fractures. © 2019 The Authors. *JBMR Plus* published by Wiley Periodicals, Inc. on behalf of American Society for Bone and Mineral Research.

## Introduction

Osteoporosis is a metabolic skeletal disease characterized by low bone mass and microarchitecture deterioration that frequently affects older adults.^1^, ^2^ The clinical consequence of osteoporosis is the occurrence of low‐impact fractures, resulting in increased mortality, morbidity, and disability, as well as imposing a major economic burden on European health care systems.^3^, ^4^ One strategy for preventing osteoporosis‐related fractures is to refine tools for identifying individuals with a high risk of fracture, as almost half of all fractures occur in individuals who are not classified as high risk by DXA scanning.^5^ To improve fracture risk assessment, several algorithms have been developed and validated.^6^ These include clinical risk factors (CRFs) such as age, gender, BMI, prior fragility fracture, parental history of hip fracture, use of oral glucocorticoids, rheumatoid arthritis, and other secondary causes of osteoporosis, including current smoking and alcohol intake to predict low‐impact fracture, independently of BMD.^7^ Although the performance of these fracture risk‐prediction tools is good, there is room for improvement in sensitivity and specificity.^6^, ^8^ The clinical challenge faced today is the accurate selection of individuals with a high risk of fracture and with indication for treatment to minimize individual and societal costs.^1^, ^5^

Serum assays for biochemical markers are important for monitoring alterations in bone formation and resorption, both in normal physiological conditions and in disease. Bone turnover markers (BTMs), reflecting bone remodeling,^9^ are modestly associated with fracture risk.^10^ However, uncertainty exists regarding the clinical application of BTMs, as they exhibit short‐ and long‐term within‐subject variability, and their usefulness for fracture prediction remains to be determined.^11^, ^12^ Thus, new biochemical markers of bone metabolism that better predict low‐impact fracture are needed.

Recent studies show the importance of Wnt signaling to osteoblast differentiation.^13^ The most well‐studied secreted Wnt antagonists are sclerostin (SOST), dickkopfs (DKKs), and secreted frizzled related proteins (sFRPs), which regulate osteoblast‐mediated bone formation.^14^ Some Wnt antagonists have been considered not only as treatment targets,^15^ but also as potential markers of bone fragility. Serum levels of DKK1 and SOST increase with age and are associated with low bone mass.^16^, ^17^ sFRP‐1 overexpression decreases bone density and attenuates the bone anabolic effects of PTH.^18^ DKK2 can either behave as a Wnt agonist or antagonist, depending on the cellular context.^19^ DKK2 inhibits bone formation in the absence of Wnt7b, but induces terminal osteoblast differentiation in the presence of high Wnt7b levels.^20^ Also, DKK2‐null mice are osteopenic with suppressed bone formation.^21^ Currently, however, there are conflicting results regarding the association between serum levels of DKK1 and SOST and low‐impact fractures.^22^, ^23^, ^24^, ^25^, ^26^ In addition, to the best of our knowledge, no studies have addressed the associations of DKK2 and sFRP‐1 with fracture.

The aim of the present study was to evaluate the association of serum levels of SOST, DKK1, DKK2, and sFRP‐1 with BMD, and the incidence of low‐impact fractures in elderly women from a population‐based cohort.

## Subjects and Methods

### Participants

This study was conducted as part of the Epidemiology of Chronic Diseases (EpiDoC) Cohort initiated in 2011. EpiDoC is a prospective closed cohort study based on a nationally representative sample of adults (≥18 years old) who were noninstitutionalized and living in private households in Portugal Mainland and Islands (Azores and Madeira). The primary aim of the baseline assessment EpiDoC 1 (EpiReumaPt), which occurred between September 2011 and December 2013, was to assess rheumatic and musculoskeletal disease prevalence and burden in Portugal. Multistage random sampling was used for participant selection. Baseline assessment consisted of two phases: The first phase involved a face‐to‐face interview; the second phase involved a detailed clinical evaluation of rheumatic and musculoskeletal disease performed by a rheumatologist. All participants enrolled in EpiDoC 1 (*n* = 10,661) were invited to participate in the follow‐up, of whom 10,153 (95.2%) agreed to participate.

For follow‐up waves EpiDoC 2 (2013 to 2015) and EpiDoC 3 (2015 to 2016), data were collected using a structured questionnaire through phone interviews using a computer‐assisted personal interview system. In each follow‐up interview, research assistants applied a nuclear questionnaire (including questions on new rheumatic disease onset, new fragility fractures, falls, medical treatment, and hospitalizations) and additional questions for each wave depending on its focus. When a contact was not available, further attempts were made at varying timepoints (eg, mornings, afternoons, evenings, and weekends) for a total of six attempts. Attempts were abandoned only when the last contact attempt occurred at least 1 month after the previous contact. Rescheduling of the telephone interview was permitted.

Necessary sample size was calculated considering the primary aim of EpiDoC 1, which was to determine the prevalence of rheumatoid arthritis with 95% CIs standardized for age and gender according to the total adult population of the studied areas. Assuming an expected prevalence of rheumatoid arthritis of 0.5% to 1% and a dropout rate of 50%, 9000 participants needed to be recruited. We recruited 10,661 participants.

#### Study population

The population of interest for the present study was women aged ≥65 years who were observed by rheumatologists during the second phase of the baseline EpiDoC 1 assessment and agreed to be followed up in subsequent EpiDoC waves. A full description of this population is provided elsewhere.^27^ Women under osteoporosis treatment or diagnosed with bone metastasis or other bone metabolic diseases, such as Paget's disease of bone, were excluded from this study.

### Outcome definition and assessment

Fragility fracture events were defined as any self‐reported low‐impact fracture occurring after 40 years of age, including fractures resulting from a fall from a standing height or sustained fractures in the absence of trauma.^28^, ^29^ Self‐reports of fragility fractures have been shown to be accurate.^30^, ^31^, ^32^ Incident fractures were defined as self‐reported new fractures during the two follow‐up waves. The follow‐up period was computed as time from baseline visit to the report of the first fracture, death, or the planned study ending, whichever occurred first.

### Covariate definition and assessment

CRFs for fracture including age, BMI (calculated using self‐reported weight and height and categorized as underweight = <18.5 kg/m^2^, normal weight = 18.5 to 24.9 kg/m^2^, overweight = 25 to 29.9 kg/m^2^, obese = ≥30 kg/m^2^), parental history of hip fracture, long‐term use of oral glucocorticoids (≥3 months), rheumatoid arthritis, current smoking, high alcohol intake (≥3 units/day), and number of falls in the previous 12 months were collected at baseline. Secondary osteoporosis was identified if the participant had a disorder strongly associated with osteoporosis. These include type I (insulin dependent) diabetes, osteogenesis imperfecta in adults, untreated long‐standing hyperthyroidism, hypogonadism or premature menopause (<45 years), chronic malnutrition, or malabsorption and chronic liver disease.

Self‐reported previous fragility fractures (ie, prevalent fragility fractures) were also recorded at baseline. Ten‐year probability of major hip fracture was calculated using the Fracture Risk Assessment (FRAX) tool^33^ without using hip DXA information. Physical activity level was classified based on the self‐reported weekly frequency of physical activity: inactive = <1 hour/week and active = ≥1 hour/week.

### DXA procedure

All women aged ≥65 years who attended the second phase of the baseline assessment were invited to undergo lumbar and nondominant hip BMD measurement (g/cm^2^) using DXA scanning (Hologic QDR 4500 A; Hologic, Bedford, MA, USA). Quality control procedures were performed according to the manufactureŕs recommendations.

### Biochemical assessment

Blood samples were collected at baseline.^34^ Serum was separated by centrifugation (800*g* for 10 min at room temperature) and kept at 4 °C. Serum samples were sent to a central diagnostic laboratory to determine levels of bone remodeling markers, 25‐hydroxyvitamin D_3_, intact PTH, and creatinine. The remaining samples were stored at −80 °C at Biobanco‐IMM (Lisbon Academic Medical Centre, Lisbon, Portugal).^34^, ^35^

At the central lab, parameters were measured according to manufacturers’ instructions. Serum levels of creatinine were measured using the rate‐blanked creatinine method (Dimension Vista Intelligent Lab System; Siemens Healthcare, Erlangen, Germany), and the glomerular filtration rate was calculated.^36^ Serum levels of PTH, osteocalcin, crosslinked C‐telopeptide of type I collagen (CTX‐I), and amino‐terminal propeptides of type I procollagen (P1NP) were measured using a fully automated Immulite 2000 electrochemiluminescent immunoassay analyzer (Siemens Healthcare). Serum levels of 25‐hydroxyvitamin D_3_ were measured using competitive immunoassay (Liason Analyzer; DiaSorin, Saluggia, Italy).

### Measurement of Wnt signaling pathway regulators

Wnt signaling regulators were measured in serum stored at Biobanco‐IMM^35^ in a subsample of women randomly selected. Baseline serum levels of sFRP‐1 (Cloud‐Clone Corp., Katy, TX, USA; intra‐assay coefficient of variation [CV] <10%; interassay CV <12%), DKK2 (Elabscience, Wuhan, China; intra‐assay CV <7%; interassay CV <7%), DKK1 (Biomedica Medizinprodukte, Vienna, Austria; intra‐assay CV ≤3%; interassay CV <3%), and SOST (Biomedica Medizinprodukte; intra‐assay CV ≤7%; interassay CV ≤10%) were determined by commercially available ELISA according to the manufacturers’ instructions and were analyzed using a Tecan Infinite 200 PRO plate reader (Tecan, Männedorf, Switzerland).

### Statistical analysis

Data are presented as mean ± SD or frequency and proportion unless stated otherwise. Baseline characteristics of participants with and without incident fragility fracture were compared using univariable logistic regression analysis. Associations between serum levels of Wnt signaling regulators (sFRP‐1, DKK2, DKK1, and SOST) were analyzed using Pearson correlations. Associations between serum levels of Wnt signaling regulators and continuous or *T*‐score categories of axial BMD (lumbar and nondominant hip) were also analyzed using Pearson correlations. Associations between serum levels of Wnt signaling regulators and BMD were analyzed by univariable linear regression and adjusted for age, BMI, family history of hip fracture, physical activity, and glucocorticoid use. Associations between serum levels of Wnt signaling regulators and incident fragility fracture were analyzed using Cox's proportional hazard models with serum levels of Wnt signaling regulators as continuous or standardized (per 1 SD) measures. Fracture risk estimates were adjusted for age, family history of hip fracture, and prevalent fragility fracture. Adjustment for lumbar and hip BMD was performed in a separate Cox regression model.

To further identify high‐risk subgroups of women for incident fragility fracture, serum levels of DKK2 were categorized into quartiles and sFRP‐1 were categorized into quintiles. To classify exposure (serum levels of DKK2 and of s‐FRP‐1) into two categories, cutpoint selection was decided using a “outcome‐oriented approach” as described by Schulgen and colleagues.^37^ The relationship between DKK2 (ng/mL) quartile (Q1, Q2 versus Q3, Q4) and incident fracture rate (per 1000 person‐years) was assessed and adjusted for age, family history of hip fracture, prevalent fragility fracture, and hip BMD. The relationship between sFRP‐1 (ng/mL) quintile (Q1, Q2 versus Q3, Q4, Q5) and incident fracture rate (per 1000 person‐years) was assessed and adjusted for age, family history of hip fracture, and prevalent fragility fracture. Using a risk stratification approach, fracture rate (per 1000 person‐years) was calculated considering the combination of 10‐year risk of major fracture Portuguese cutoff (<11% versus ≥11%) without BMD and serum level of DKK2 (lowest two quartiles versus highest two quartiles). The FRAX score cutoff was based on the Portuguese recommendation for fracture risk prediction.^38^

Statistical significance was established as *p <* 0.05. All analyses were performed using Stata IC, version 12 (StataCorp LP, College Station, TX, USA).

### Ethics approval

The EpiDoC Cohort study was approved by the Ethics Committee of Nova Medical School (Lisbon, Portugal) and the Portuguese Data Protection Authority (Comissão Nacional de Proteção de Dados, Lisbon, Portugal). Written informed consent in accordance with principles established by the Declaration of Helsinki was obtained from all participants. Further details related to ethical issues are described elsewhere.^39^

## Results

**Figure 1:**
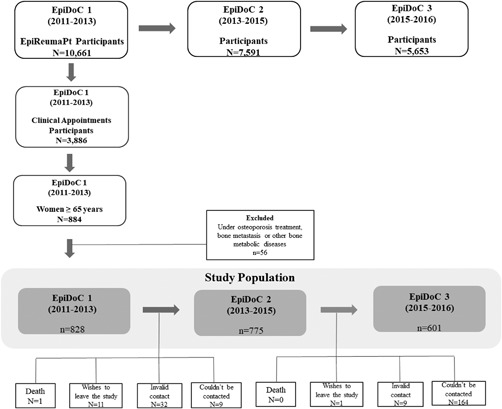
Flowchart of study participants.

Of 3877 participants evaluated by a rheumatologist at baseline, 884 were women aged ≥65 years. After applying exclusion criteria, 828 women were included in this study (Fig. 1). A small proportion of women (*n* = 71; 8.57%) had received bisphosphonates in the past. During a mean follow‐up of 2.3 ± 1.0 years, 62 fragility fractures were sustained in 58 women. Most incident fragility fractures (*n* = 51; 82.3%) were nonhip, nonvertebral (ie, wrist, lower leg, humerus, rib, clavicle, and elbow). Incident hip or vertebral fractures were reported by 6 (9.7%) and 4 (6.6%) women, respectively. Senior women with incident fragility fractures had significantly more prior fractures and had a family history of hip fractures more frequently. No other CRFs were associated with incident fragility fracture (Table 1).

**Table 1:** Crude Analysis of Sociodemographic and Economic Characteristics, Risk Factors for Fractures, and Health Status of Portuguese Women Aged ≥65 Years With or Without Incident Fragility Fractures

	All (*n* = 828)	No incident fragility fracture (*n* = 669)	Incident fragility fracture (*n *= 58)	*p* value
Age (years)				
65–69	270 (32.61%)	232 (34.68%)	17 (29.31%)	0.277
70–79	414 (50.00%)	339 (50.67%)	28 (48.28%)	
≥80	144 (17.39%)	98 (14.65%)	13 (22.41%)	
BMI (kg/m^2^)				
Underweight	7 (0.86%)	5 (0.76%)	0 (0%)	0.866
Normal	205 (25.31%)	161 (24.51%)	14 (24.14%)	
Overweight	358 (44.20%)	280 (42.62%)	28 (48.28%)	
Obese	240 (29.63%)	211 (32.12%)	16 (27.59%)	
Family history of hip fracture				
Yes	51 (6.17%)	37 (5.54%)	8 (13.79%)	0.016^a^
No	776 (93.83%)	631 (94.46%)	50 (86.21%)	
Current smoking				
Yes	16 (1.93%)	14 (2.10%)	1 (1.72%)	0.849
No	811 (98.07%)	654 (97.90%)	57 (98.28%)	
High alcohol intake (≥3 units/day)				
Yes	14 (1.69%)	13 (1.95%)	1 (1.72%)	0.906
No	813 (98.31%)	655 (98.05%)	57 (98.28%)	
Physical activity				
Inactive	488 (84.87%)	443 (84.70%)	43 (86.00%)	0.807
Active	87 (15.13%)	80 (15.30%)	7 (14.00%)	
Number of falls in previous 12 months	1.19 ± 3.41	1.03 ± 2.95	1.34 ± 2.27	0.450
Use of glucocorticoids				
Yes	30 (3.63%)	23 (3.44%)	4 (6.90%)	0.192
No	797 (96.37%)	645 (96.56%)	54 (93.10%)	
Rheumatoid arthritis				
Yes	13 (1.57%)	11 (1.65%)	1 (1.72%)	0.965
No	814 (98.43%)	657 (98.35%)	57 (98.28%)	
Secondary osteoporosis				
Yes	25 (3.02%)	20 (2.99%)	3 (5.17%)	0.370
No	802 (96.98%)	648 (97.01%)	55 (94.83%)	
Chronic renal insufficiency (mL/min/1.73 m^2^)				
eGFR <30	16 (2.51%)	13 (2.50%)	2 (4.76%)	0.392
eGFR ≥30	621 (97.49%)	506 (97.50%)	40 (95.24%)	
Prevalent fragility fracture (self‐reported)				
Yes	172 (21.83%)	121 (18.94%)	26 (45.61%)	<0.001^a^
No	616 (78.17%)	518 (81.06%)	31 (54.39%)	
Prevalent fragility fracture site (self‐reported)				
Hip	10 (1.27%)	4 (0.63%)	2 (3.51%)	0.046^a^
Vertebral	11 (1.40%)	7 (1.10%)	2 (3.51%)	0.144
Nonhip/nonvertebral	121 (15.94%)	86 (13.85%)	16 (32.00%)	0.001^a^
FRAX score without BMD				
10‐year risk of major fracture (mean ± SD)	9.62 ± 6.85	9.00 ± 6.04	12.83 ± 10.71	<0.001^a^
10‐year risk of hip fracture (mean ± SD)	4.24 ± 5.20	3.79 ± 4.29	6.64 ± 9.78	<0.001^a^
Lumbar spine BMD (g/cm^2^)				
Lumbar spine BMD (mean ± SD)	0.99 ± 0.21	1.00 ± 0.21	0.97 ± 0.21	0.471
Lumbar spine BMD (*T* score)				
Osteoporosis (≤−2.5)	69 (25.75%)	55 (23.81%)	6 (35.29%)	0.517
Osteopenia (>−2.5 and <−1)	89 (33.21%)	78 (33.77%)	4 (23.53%)	
Normal (≥−1)	110 (41.04%)	98 (42.42%)	7 (41.18%)	
Axial BMD (*T* score)				
Osteoporosis (≤−2.5)	74 (27.31%)	59 (25.32%)	7 (38.89%)	0.398
Osteopenia (>−2.5 and <−1)	128 (47.23%)	111(47.64%)	6 (33.33%)	
Normal (≥−1)	69 (25.46%)	63 (27.04%)	5 (27.78%)	
Hip BMD (g/cm^2^)				
Hip BMD (mean ± SD)	0.78 ± 0.14	0.78 ± 0.14	0.79 ± 0.12	0.865
Hip BMD (*T* score)				
Osteoporosis (≤−2.5)	27 (9.93%)	21 (8.97%)	3 (16.67%)	0.464
Osteopenia (>−2.5 and <−1)	139 (51.10%)	119 (50.85%)	7 (38.89%)	
Normal (≥−1)	106 (38.97%)	94 (40.17%)	8 (44.44%)	
Vitamin D (mmol/mL)				
Deficiency (<10)	18 (2.96%)	15 (3.02%)	3 (7.50%)	0.341
Insufficiency (≥10 and <30)	212 (34.81%)	175 (35.28%)	13 (32.50%)	
Normal (≥10)	379 (62.23%)	306 (61.69%)	24 (60.00%)	
Bone turnover markers				
CTX‐I (ng/mL)	0.25 ± 0.16	0.25 ± 0.16	0.27 ± 0.21	0.612
P1NP (ng/mL)	41.06 ± 20.50	39.75 ± 18.36	41.39 ± 20.06	0.716
Osteocalcin (ng/mL)	3.77 ± 2.46	3.60 ± 2.28	4.05 ± 1.85	0.412
PTH (ng/mL)	49.38 ± 38.69	47.69 ± 36.45	50.24 ± 46.56	0.681
Serum levels of Wnt regulators				
DKK2 (ng/mL)	7.79 ± 2.86	7.75 ± 2.75	6.86 ± 2.39	0.144
sFRP‐1 (ng/mL)	2.02 ± 1.37	1.92 ± 1.31	2.56 ± 1.37	0.035^a^
SOST (pmol/L)	31.89 ± 13.96	32.14 ± 13.80	30.91 ± 15.64	0.700
DKK1 (pmol/L)	132.24 ± 76.48	136.76 ± 76.74	118.16 ± 81.00	0.311

Sample size is not constant. All: BMI (*n* = 810), family history of hip fracture (*n* = 827), current smoking (*n* = 827), high alcohol intake (*n* = 827), physical activity (*n* = 575), number of falls in previous 12 months (*n* = 784), glucocorticoid use (*n* = 827), rheumatoid arthritis (*n* = 827), secondary osteoporosis (*n* = 827), chronic renal insufficiency (*n* = 637), prevalent fragility fracture (*n* = 788), hip (*n* = 788), vertebral (*n* = 788), nonvertebral/nonhip (*n* = 759), prevalent vertebral fracture (*n* = 318), FRAX (Fracture Risk Assessment Tool) major (*n* = 820), FRAX hip (*n* = 820), lumbar spine BMD (*n* = 268), hip BMD (*n* = 271), vitamin D (*n* = 609), CTX‐I (*n* = 289), P1NP (*n* = 287), osteocalcin (*n* = 291), PTHi (*n* = 592), SOST (*n* = 321), DKK1 (*n* = 290), DKK2 (*n* = 319), sFRP1 (*n* = 321). No incident fragility fracture: BMI (*n* = 657), family history of hip fracture (*n* = 668), current smoking (*n* = 668), high alcohol intake (*n* = 668), physical activity (*n* = 523), number of falls in previous 12 months (*n* = 634), glucocorticoid use (*n* = 668), rheumatoid arthritis (*n* = 668), secondary osteoporosis (*n* = 668), chronic renal insufficiency (*n* = 519), prevalent fragility fracture (*n* = 639), hip (*n* = 639), vertebral (*n* = 639), nonvertebral/nonhip (*n* = 621), prevalent vertebral fracture (*n* = 276), FRAX major (*n* = 663), FRAX hip (*n* = 663), lumbar spine BMD (*n* = 231), hip BMD (*n* = 233), vitamin D (*n* = 496), CTX‐I (*n* = 241), P1NP (*n* = 241), osteocalcin (*n* = 243), PTHi (*n* = 481), SOST (*n* = 277), DKK1 (*n* = 249), DKK2 (*n* = 275), sFRP‐1 (*n* = 276). Incident fragility fracture: physical activity (*n* = 50), number of falls in previous 12 months (*n* = 56), chronic renal insufficiency (*n* = 42), prevalent fragility fracture (*n* = 57), hip (*n* = 57), vertebral (*n* = 57), nonvertebral/nonhip (*n* = 50), prevalent vertebral fracture (*n* = 21), FRAX major (*n* = 57), FRAX hip (*n* = 57), lumbar spine BMD (*n* = 17), hip BMD (*n* = 18), vitamin D (*n* = 40), CTX‐I (*n* = 18), P1NP (*n* = 18), osteocalcin (*n* = 18), PTHi (*n* = 39), SOST (*n* = 20), DKK1 (*n* = 19), DKK2 (*n* = 21), sFRP1 (*n* = 21).

^a^ *p* < 0.05.

### Serum levels of sFRP‐1, SOST, and DKK1 are associated with BMD

There was a negative correlation between baseline serum levels of DKK1 and DKK2 (*r* = −0.152; *p* = 0.01). No other correlations were found among serum levels of Wnt regulators (Supplementary Table S1).

There was no correlation between serum levels of DKK2 and BMD (Table 2). Serum levels of sFRP‐1 and SOST were positively correlated with lumbar and hip BMD (Table 2). When BMD was categorized by *T* score, the positive correlations for both sFRP‐1 and SOST were lost, except in the normal BMD group. After adjusting for age, BMI, family history of hip fracture, physical activity, and glucocorticoid use, the serum level of sFRP‐1 was positively correlated with lumbar spine BMD (β = 0.040, *p* < 0.001) and hip BMD (β = 0.011, *p* = 0.001; Supplementary Table S2). Using the same adjustment parameters, SOST levels were still positively correlated with lumbar spine BMD (β = 0.001, *p* < 0.001) and negatively correlated with hip BMD (β = −0.002, *p* = 0.001; Supplementary Table S2).

**Table 2:** Correlations Between Serum Levels of DKK2, SOST, DKK1, and sFRP‐1, and BMD Stratified by *T*‐Score Groups

	Lumbar spine BMD (g/cm^2^)				Hip BMD (g/cm^2^)			
	Continuous (g/cm^2^)	Osteoporosis (*T* score ≤−2.5)	Osteopenia (*T* score >−2.5 and <−1)	Normal (*T* score ≥−1)	Continuous (g/cm^2^)	Osteoporosis (*T* score ≤−2.5)	Osteopenia (*T* score >−2.5 and <−1)	Normal (*T* score ≥−1)
DKK2 (ng/mL)	−0.0537	−0.1415	0.0774	0.0042	−0.0279	0.0081	0.0194	−0.0034
sFRP−1 (ng/mL)	0.2603^c^	0.0652	0.1864	0.4066^c^	0.1621^a^	0.0223	0.1912	0.1172
SOST (pmol/L)	0.2944^c^	−0.1569	0.0591	0.2099^a^	0.1917^b^	0.0244	0.1781	0.0053
DKK1 (pmol/L)	−0.0162	0.1610	−0.2916^a^	−0.0385	−0.1725^b^	−0.1662	−0.2235^a^	−0.2083

Sample size is not constant. Lumbar spine BMD: SOST (*n* = 245), DKK1 (*n* = 220), DKK2 (*n* = 243), sFRP‐1 (*n* = 244). Hip BMD: SOST (*n* = 247), DKK1 (*n* = 218), DKK2 (*n *= 245), sFRP1 (*n* = 246).

^a^ *p* < 0.05.

^b^ *p* < 0.01.

^c^ *p* < 0.001.

By contrast, serum levels of DKK1 were negatively correlated with hip femoral neck BMD. This association remained significant even after adjusting for CRFs (β = −0.0004, *p* = 0.008; Supplementary Table S2).

### Serum levels of DKK2 and sFRP‐1 are independently associated with incident low‐impact fracture

**Figure 2:**
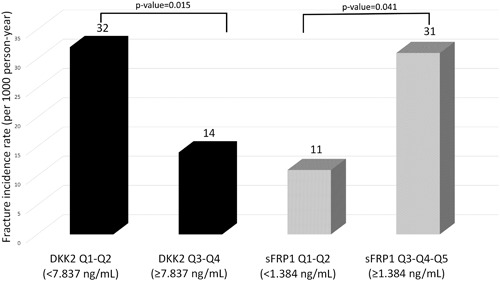
Association between DKK2 and sFRP‐1 quartiles with incident fracture rate (per 1000 person‐years). Women in the two highest DKK2 quartiles had a significantly lower fracture incidence than women in the two lowest DKK2 quartiles. Women in the two lowest sFRP‐1 quartiles had significantly lower fracture incidence than women in the two highest sFRP‐1 quartiles.

Low serum level of DKK2 was associated with an increased risk of low‐impact fracture in Cox proportional hazard models (Table 3). This association remained significant after adjusting for independent CRFs for low‐impact fracture identified in this population as age, family history of hip fracture, and prevalent fragility fracture measured as HR (95% CI) per 1 SD increase (HR 0.61; 95% CI, 0.39 to 0.98). The HR also remained significant after adjusting for lumbar spine BMD measured as HR (95% CI) per 1 SD increase (HR 0.47; 95% CI, 0.27 to 0.82) and hip BMD measured as HR (95% CI) per 1 SD increase (HR 0.53; 95% CI, 0.32 to 0.88; Table 3). Women in the two highest DKK2 quartiles had a fracture incidence rate of 14 per 1000 person‐years, whereas women in the two lowest DKK2 quartiles had a fracture incidence rate of 32 per 1000 person‐years (Fig. 2).

**Table 3:** Crude and Adjusted Analysis of Associations Between Serum Levels of Wnt Regulators and Bone Turnover Markers and Incident Fragility Fracture

Incidence fracture	Continuous		Per 1 SD increase	
**WNT regulators**				
**DKK2 (ng/mL)**	**HR**	***p***	**HR**	***p***
Crude	0.861 (0.738; 1.006)	0.059	0.655 (0.423; 1.016)	0.059
CRFs adjusted^1^	0.842 (0.714; 0.993)	0.041^a^	0.615 (0.386; 0.979)	0.041^a^
CRFs + lumbar spine BMD adjusted^2^	0.767 (0.630; 0.933)	0.008^a^	0.471 (0.271; 0.821)	0.008^a^
CRFs + hip BMD adjusted^3^	0.798 (0.665; 0.958)	0.015^a^	0.529 (0.316; 0.885)	0.015^a^
**sFRP‐1 (ng/mL)**	**HR**	***p***	**HR**	***p***
Crude	1.318 (1.008; 1.722)	0.043^a^	1.453 (1.011; 2.087)	0.043^a^
CRFs adjusted^1^	1.431 (1.067; 1.918)	0.017^a^	1.624 (1.092; 2.416)	0.017^a^
CRFs + lumbar spine BMD adjusted^2^	1.265 (0.891; 1.796)	0.188	1.375 (0.856; 2.209)	0.188
CRFs + hip BMD adjusted^3^	1.329 (0.971; 1.819)	0.075	1.470 (0.961; 2.248)	0.075
**SOST (pmol/L)**	**HR**	***p***	**HR**	***p***
Crude	1.001 (0.970; 1.033)	0.939	1.017 (0.661; 1.564)	0.939
CRFs adjusted^1^	1.007 (0.975; 1.039)	0.677	1.097 (0.709; 1.696)	0.677
CRFs + lumbar spine BMD adjusted^2^	0.999 (0.957; 1.043)	0.956	0.983 (0.545; 1.774)	0.956
CRFs + hip BMD adjusted^3^	0.989 (0.949; 1.030)	0.578	0.853 (0.488; 1.491)	0.578
**DKK1 (pmol/L)**	**HR**	***p***	**HR**	***p***
Crude	0.998 (0.990; 1.003)	0.512	0.845 (0.511; 1.396)	0.512
CRFs adjusted^1^	0.996 (0.989; 1.004)	0.307	0.743 (0.421; 1.313)	0.307
CRFs + lumbar spine BMD adjusted^2^	0.993 (0.983; 1,003)	0.151	0.588 (0.285; 1.214)	0.151
CRFs + hip BMD adjusted^3^	0.991 (0.982; 1.000)	0.078	0.510 (0.241; 1.079)	0.078
**Bone turnover markers**				
**CTX‐I (ng/mL)**	**HR**	***p***	**HR**	***p***
Crude	2.076 (0.197; 21.862)	0.543	1.126 (0.768; 1.652)	0.543
CRFs adjusted^1^	2.687 (0.241; 29.973)	0.422	1.175 (0.793; 1.740)	0.422
CRFs + lumbar spine BMD adjusted^2^	1.469 (0.001; 3849.7)	0.924	1.065 (0.295; 3.836)	0.924
CRFs + hip BMD adjusted^3^	1.024 (0.001; 1544.9)	0.995	1.004 (0.305; 3.306)	0.995
**P1NP (ng/mL)**	**HR**	***p***	**HR**	***p***
Crude	1.005 (0.981; 1.030)	0.679	1.111 (0.679; 1.813)	0.679
CRFs adjusted^1^	1.010 (0.985; 1.035)	0.434	1.217 (0.744; 1.992)	0.434
CRFs + lumbar spine BMD adjusted^2^	1.032 (0.965; 1.104)	0.361	1.885 (0.484; 7.346)	0.361
CRFs + hip BMD adjusted^3^	1.025 (0.966; 1.088)	0.413	1.654 (0.496; 5.521)	0.413
**Osteocalcin (ng/mL)**	**HR**	***p***	**HR**	***p***
Crude	1.075 (0.925; 1.250)	0.346	1.191 (0.828; 1.713)	0.346
CRFs adjusted^1^	1.091 (0.936; 1.272)	0.266	1.234 (0.852; 1.787)	0.266
CRFs + lumbar spine BMD adjusted^2^	1.299 (0.963; 1.751)	0.087	1.879 (0.913; 3.868)	0.913
CRFs + hip BMD adjusted^3^	1.123 (0.870; 1.450)	0.373	1.324 (0.714; 2.452)	0.373

^1^ Adjusted for age, family history of hip fracture, and prevalent fragility fracture (self‐reported).

^2^ Adjusted for age, family history of hip fracture, prevalent fragility fracture (self‐reported), and lumbar spine BMD.

^3^ Adjusted for age, family history of hip fracture, prevalent fragility fracture (self‐reported), and hip BMD. Sample size is not constant. SOST: crude (*n* = 529), adjusted^1^ (*n* = 496), adjusted^2^ (*n* = 368), adjusted^3^ (*n* = 371). DKK1: crude (*n* = 476), adjusted^1^ (*n* = 443), adjusted^2^ (*n* = 323), adjusted^3^ (*n* = 320). DKK2: crude (*n* = 527), adjusted^1^ (*n* = 494), adjusted^2^ (*n* = 366), adjusted^3^ (*n* = 369). sFRP‐1: crude (*n* = 529), adjusted^1^ (*n* = 496), adjusted^2^ (*n* = 366), adjusted^3^ (*n* = 369). CTX‐I: crude (*n* = 450), adjusted^1^ (*n* = 420), adjusted^2^ (*n* = 138), adjusted^3^ (*n* = 138). P1NP: crude (*n* = 451), adjusted^1^ (*n* = 421), adjusted^2^ (*n* = 138); adjusted^3^ (*n* = 140). Osteocalcin: crude (*n* = 455), adjusted^1^ (*n* = 425), adjusted^2^ (*n* = 138), adjusted^3^ (*n* = 140). CRFs = clinical risk factors.

^a^ *p* < 0.05.

A high serum level of sFRP‐1 was associated with an increased risk of low‐impact fracture measured as HR (95% CI) per 1 SD increase (HR 1.45; 95% CI, 1.01 to 2.09). This association was independent of CRFs measured as HR (95% CI) per 1 SD increase (HR 1.62; 95% CI, 1.09 to 2.42), but dependent on BMD (Table 3). Women in the two lowest sFRP‐1 quartiles had a fracture incidence rate of 11 per 1000 person‐years, whereas women in the two highest sFRP1 quartiles had a fracture incidence rate of 31 per 1000 person‐years (Fig. 2).

Cox proportional hazard models showed no association between serum level of SOST or DKK1 and fracture risk in senior women. Also, no associations were found between BTMs and low‐impact fracture incidence (Table 3).

### FRAX score accurately predicts incident low‐impact fractures

Women designated at high fracture risk using FRAX score (women with a 10‐year risk of major fracture ≥11%)^38^ had a higher fracture incidence rate than women designated as low fracture risk (women with a 10‐year risk of major fracture <11%; 41 per 1000 person‐years versus 18 per 1000 person‐years; *p* = 0.002) In fact, women with a 10‐year risk of major fracture ≥11% had a 2.4‐fold increased risk of low impact fractures (HR 2.37; 95% CI, 1.40 to 4.00) compared with women with a 10‐year risk of major fracture <11%.

### Serum level of DKK2 improves fracture risk prediction independently of FRAX score

**Figure 3:**
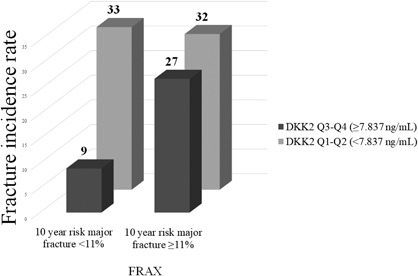
Relationship between DKK2 quartiles and Fracture Risk Assessment (FRAX) tool score. Women in the two lowest DKK2 quartiles had the highest fracture incidence rate independently of being under or above the cutoff for 10‐year major fracture risk (ie, 11%).

Using a risk‐stratified approach, the fracture incidence rate (per 1000 person‐years) was calculated based on the combination baseline 10‐year risk of major fracture using FRAX score (without BMD) and serum level of DKK2 (Fig. 3). Low serum levels of DKK2 can enhance fracture risk prediction in women considered as low risk of fracture by FRAX score. Women with low serum levels of DKK2 (33 per 1000 person‐years) had a higher fracture incidence rate than women with high serum levels of DKK2 (9 per 1000 person‐years; *p* < 0.038; Fig. 3).

## Discussion

The present study, conducted in a population‐based cohort of senior women, showed that low serum level of DKK2 predicted low‐impact fractures, independently of BMD and CRFs for fracture. For every 1 SD decrease in DKK2, fracture risk increased by approximately 1.5‐fold. Serum levels of DKK2 were not associated with lumber spine or hip BMD. Our results need to be further investigated in a larger study with a longer follow‐up period.

DKK molecules are Wnt‐β‐catenin signaling regulators.^40^ DKK2 is mainly expressed by osteoblasts and has a dual effect on Wnt‐β‐catenin signaling. It is a potent antagonist of Wnt‐β‐catenin signaling, by binding to low‐density lipoprotein receptor‐related proteins 5 and 6, and by controlling osteoblast differentiation. However, it also has a weak agonistic effect. Overall, DKK2 acts as a fine‐tuning regulator of osteoblast terminal differentiation and function through Wnt signaling dependent and independent mechanisms, leading to an enhancement of mineralization.^20^ DKK2 is upregulated in osteoarthritis subchondral bone (with local high bone mass); in vitro upregulation of DKK2 in osteoblasts increases their ability to form mineralized nodules.^41^ By contrast, DKK2 deficiency in vivo leads not only to mineralization disturbances and bone fragility, but also to a moderate reduction in bone mass.^20^ These results are in line with our present findings showing that a decreased serum level of DKK2 was associated with bone fragility fracture, but not with lumbar spine and hip BMD. This may be because osteoblast mineralization disturbances, signalled by low levels of DKK2, lead to bone nanoarchitecture disorganization and fragility, independently of bone mass loss.^42^, ^43^, ^44^

DKK1 is a key inhibitor of LRP5/6 and of Wnt‐β‐catenin signaling, and is mainly expressed by osteocytes. It is therefore expected that an increased DKK‐1 expression will be associated with a decreased Wnt activity and bone mass.^14^ In line with that, we observed a negative correlation between serum levels of DKK1 and BMD, similar to what was previously reported.^45^, ^46^ However, we found no association between DKK1 serum levels and incident fracture. In line with our results, a study from Korea did not find an association of serum levels of DKK1 and prevalent osteoporotic fractures.^25^ In contrast, in a cross‐sectional study in Sweden, serum levels of DKK1 were increased in patients with a fresh hip fracture when compared with healthy volunteers.^47^

sFRP‐1 and SOST are also Wnt‐β‐catenin signaling inhibitors. Overexpression of sFRP‐1 in human osteoblasts accelerates the rate of cell death, and thus inhibits bone formation.^18^, ^48^ Concordantly, we found that high levels of sFRP‐1 were associated with incident low‐impact fracture, independent of CRFs for fracture. However, this association was not independent of BMD. Surprisingly, in our study, high serum levels of sFRP‐1 and SOST were significantly associated with high lumbar spine and hip BMD. Given that sFRP‐1 and SOST inhibit osteoblast proliferation and maturation, we would have expected a negative correlation with BMD. Other studies also found the same paradoxical results.^16^, ^23^, ^49^ Of interest, when we analyzed the association between serum levels of SOST and sFRP‐1 and BMD categorized by *T* score, we confirmed the existence of correlations in women with normal BMD, but not with osteopenia or osteoporosis. One possible explanation is that in healthy individuals, normal BMD is maintained through local downregulation of Wnt inhibitors in association with high systemic serum levels of SOST and sFRP‐1. In agreement with this possibility, the serum level of SOST is higher in men (who have a lower global fracture risk) than in women.^50^ However, in pathological situations, as in individuals with bone fragility and a high risk of fracture, this regulation system is disrupted, and serum levels of Wnt regulators are associated with osteoblast dysfunction, bone fragility, and fracture, as our results showed.

In our study, serum levels of SOST were not significantly associated with incident low‐impact fracture. Similarly, the OFELY study followed 572 postmenopausal women for 6 years and reported no association between serum level of SOST with incident nonvertebral and clinical vertebral fractures.^22^ Amrein and colleagues also found no association between SOST serum level with hip and other nonvertebral fractures in institutionalized elderly women.^51^ By contrast, the Center of Excellence for Osteoporosis Research Study followed 707 postmenopausal women for 5 years and showed that a high serum SOST level is associated with an increased risk of vertebral and nonvertebral low‐impact fracture.^24^ Concordantly, Arasu et al. also found that serum levels of SOST were associated with hip fractures among women aged 65 years or older.^23^

We also found no associations between serum levels of BTMs (P1NP, CTX‐I, and osteocalcin) and fragility fractures. Although several studies have proposed BTMs as fracture risk predictors, their results are not conclusive.^10^, ^52^, ^53^, ^54^, ^55^ A recent meta‐analysis reports a modest association between CTX‐I and fragility fracture, although this association is not independent of BMD.^10^ These ambiguous study results probably contribute to the low acceptance and utility of BTMs in clinical practice and re‐enforce the need to find alternative markers of fragility fracture risk.

This study has some limitations. First, fragility fractures were self‐reported, which is less accurate than clinically verified vertebral fractures, leading to the underestimation of their prevalence^56^; however, the overall performance of self‐reported fragility fractures is respectable.^30^, ^31^, ^32^ Second, the number of incident fractures was relatively low because of a short follow‐up duration (2.3 ± 1.0 years). DKK2 were measured in a randomized subsample (*n* = 319) of our population (*N* = 828) and 19 incident fractures were recorded in this subsample. Hence, these results must be confirmed in other cohorts with more participants and longer follow‐up periods. Nevertheless, several strengths of this study should also be acknowledged. Our data came from a large, representative sample of the Portuguese adult population and participants were examined by rheumatologists at baseline. Furthermore, different fragility fractures and health‐related measurements were captured, providing relevant information about risk factors.

In conclusion, we report that low serum levels of DKK2 predict the risk of low‐impact fractures, independently of BMD and CRFs and thus should be explored as a potential noninvasive marker of fragility fracture risk. High serum levels of sFRP‐1 were significantly associated with fracture, although this association was not independent of BMD. Both SOST and DKK1 were associated with BMD, but not with incident fracture, although the number of new fractures recorded may not have allowed the detection of these latter associations. These results indicate that serum DKK2 and sFRP1 may predict low‐impact fracture. The low number of incident fractures recorded is a limitation; hence, serum levels of Wnt regulators should be further studied in other populations as potential noninvasive markers of fragility fractures.

## Disclosures

All authors declare that they have no conflicts of interest.

## Supporting information

Supporting Tables S1.
