# Supplementary material for: Low Serum Levels of DKK2 Predict Incident Low‐Impact Fracture in Older Women
Source: JBMR Plus. 2019 Mar 28;3(7):e10179. doi: 10.1002/jbm4.10179 (PMC6659448; doi:10.1002/jbm4.10179)
Supplement: Supplementary file 1 — Supporting Tables S1. [file JBM4-3-na-s001.docx]

Suplementary Table 1. Correlations between serum levels of DKK2, SOST, DKK1, and sFRP-1

|  | DKK2 (ng/mL) | | sFRP-1 (ng/mL) | | SOST (pmol/L) | | DKK1 (pmol/L) | |
| --- | --- | --- | --- | --- | --- | --- | --- | --- |
|  | R | p-value | R | p-value | R | p-value | R | p-value |
| DKK2 (ng/mL) | - | - | -0.0397 | 0.105 | -0.0871 | 0.121 | -0.1525 | 0.01* |
| sFRP-1 (ng/mL) | -0.0397 | 0.481 | - | - | 0.0140 | 0.803 | -0.0956 | 0.105 |
| SOST (pmol/L) | -0.0871 | 0.121 | 0.0140 | 0.803 | - | - | -0.0105 | 0.859 |
| DKK1 (pmol/L) | -0.1525 | 0.01* | -0.0956 | 0.105 | -0.0105 | 0.859 | - | - |

Suplementary Table 2. Crude and adjusted analysis of associations between serum levels of DKK2, SOST, DKK1, and sFRP-1, and BMD.

|  | Lumbar spine BMD (g/cm^2^) | | | | Hip BMD (g/cm^2^) | | | |
| --- | --- | --- | --- | --- | --- | --- | --- | --- |
|  | **Crude** | | **Adjusted** | | **Crude** | | **Adjusted** | |
|  | **β** | **p** | **β** | **p** | **β** | **p** | **β** | **p** |
| DKK2 (ng/mL) | -0.004 | 0.404 | -0.003 | 0.536 | -0.002 | 0.664 | -0.002 | 0.613 |
| sFRP-1 (ng/mL) | 0.040 | <0.001^a^ | 0.043 | <0.001^a^ | 0.016 | 0.011^a^ | 0.016 | 0.020^a^ |
| SOST (pmol/L) | 0.005 | <0.001^a^ | 0.005 | <0.001^a^ | 0.002 | 0.002^a^ | -0.002 | 0.001^a^ |
| DKK1 (pmol/L) | -0.000 | 0.811 | -0.000 | 0.867 | -0.0003 | 0.011^a^ | -0.0004 | 0.008^a^ |

Sample size is not constant. Crude lumbar spine BMD: SOST (n=245), DKK1 (n=220), DKK2 (n=243), sFRP1 (n=244). Crude hip BMD: SOST (n=247), DKK1 (n=218), DKK2 (n=245), sFRP1 (n=246). Adjusted lumbar spine BMD: SOST (n=178), DKK1 (n=163), DKK2 (n=177), sFRP1 (n=176). Adjusted hip BMD: SOST (n=182), DKK1 (n=164), DKK2 (n=181), sFRP1 (n=180). Adjusted for age, BMI, family history of hip fracture, physical activity, and glucocorticoid use. ^a^ p<0.05.
